# The role of hyperthermia in modern radiation treatment- state of art

**DOI:** 10.1186/s13014-025-02741-5

**Published:** 2025-12-13

**Authors:** Aneta Borkowska, Paulina Chmiel, Piotr Rutkowski, Mateusz Jacek Spałek

**Affiliations:** 1 Department of Soft Tissue/Bone Sarcoma and Melanoma, Maria Sklodowska-Curie National Research Institute of Oncology, Warsaw, Poland; 2 Department of Radiotherapy I, Maria Sklodowska-Curie National Research Institute of Oncology, Warsaw, 02-718 Poland

**Keywords:** Hyperthermia, HT, Radiotherapy, RT, Immunotherapy, ITH, Radiation oncology, Cancer biology

## Abstract

The role of hyperthermia (HT) in conventional oncological treatment has been a subject of research for decades; however, HT has not been incorporated into treatment guidelines on a universal basis. Preclinical studies have demonstrated the mechanism of action of HT and have indicated a clear effect that can enhance the effects of radiotherapy (RT), chemotherapy, or immunotherapy. The underlying mechanism of HTs action involves either the enhancement of the immune system response or the interference with crucial cellular pathways that are aberrantly altered during the neoplastic process. Consequently, HT has the potential to augment the efficacy of RT treatments markedly. Randomized clinical trials have further demonstrated the efficacy and safety of combining RT and HT. However, it is important to note that the majority of these observations were derived from studies conducted up to two decades ago, which may not fully reflect the current standard of care. The present focus is on the combination of these treatment techniques with modern systemic treatment, which is based on immunotherapy and molecularly targeted drugs. Significant advancements have also been made in the field of HT delivery and the strategies for optimal use of HT. Therefore, it is imperative to synthesize the extant body of knowledge in this field to inform the advancement of techniques for integrating HT with radiation therapy.

## Introduction

Hyperthermia (HT) can be defined as an elevated tissue or body temperature that exceeds physiological levels and is induced by an external source [1]. This definition reflects the fundamental thermal mechanism that instigates physiological changes, a principle that has been employed in the domain of oncological treatment [2]. Contemporary HT employs a diverse array of techniques and temperature ranges to achieve the intended outcome. The clinical application of elevated temperatures in cancer treatment has a long history, dating back to the nineteenth century when Coley utilized preparations of killed bacterial cultures to induce fever in patients with inoperable tumors, resulting in their clinical regression, providing the foundation for development of modern HT [3]. Decades later, HT is employed as a sole treatment modality for direct tumor lesion ablation, with temperatures exceeding 50 °C [4], or as an adjunct to chemotherapy (CHT), immunotherapy (ITH), or radiotherapy (RT) in the range of 39–45 °C (mild HT), achieving superior outcomes in comparison to treatments devoid of HT [5, 6].

The combination of RT with HT has demonstrated a substantial benefit in clinical trials for various types of cancer. Noteworthy examples include breast cancer, cervical cancer, head and neck region cancers, and soft tissue sarcomas [7–10]. Mild HT, with temperatures ranging from 39 to 45 °C, is the most commonly used approach. However, it is noteworthy that this approach is not yet incorporated into standard treatment guidelines for many diagnoses, and in some cases, there is a paucity of clinical data supporting its incorporation [11–13]. Furthermore, a notable aspect of clinical trials involving the combination of RT and HT is the occurrence of adverse effects in the treated population [14]. In the context of the evolution of contemporary systemic therapies, the combination of RT with HT and immunotherapy, as well as targeted treatment, is a matter of increasing concern, particularly with respect to the systemic impact of such therapeutic regimens.

This review aims to address contemporary inquiries concerning HT and its combination with RT and its role in oncology. The review encompassed the biological basis, mechanism of action, modern techniques of heat delivery and temperature control in combination with RT, and the clinical application of this combination. A particular emphasis was placed on clinical trials currently underway that focus on this subject.

## Biological rationale for combining RT and HT

HT exerts many biological effects on the tumor, its microenvironment, and the patient’s immunological system. The majority of the biological evidence supporting the radiosensitizing effect of HT originates from preclinical studies [15–17]. These studies indicate that HT induces oxidative stress, which results in DNA damage and increased apoptosis, as well as reduces hypoxia which contributes to resistance to treatment. In addition to its strictly cellular effects, HT has been observed to affect the immune system locally and systemically. This is evidenced by the stimulation of the immune response to antigens, which is caused by an increase in their exposure. Collectively, these pathways are responsible for the beneficial effect of combining HT with RT [18]. It is therefore crucial to administer HT in a way that will induce its optimal biological effect when it is used in combination with other therapies.

### Molecular effects of hyperthermia

The impact of elevated temperature on cellular function, both in healthy and neoplastic cells, is dependent upon the temperature range utilized. When temperatures exceed 60 °C, as occurs in thermal ablations, proteins undergo a process of direct denaturation. This denaturation event initiates a series of immediate cellular responses, characterized by the onset of cell death known as coagulative necrosis [19, 20]. The utilization of lower temperatures (40–45°C) invariably introduces increased complexity to the process, often necessitating prolonged periods for heat application, however, most of those processes have a synergistic impact with RT. The phenomenon of thermal damage has been associated with mitochondrial dysfunction, a condition precipitated by aberrations in mitochondrial membranes and internal structures. It has been demonstrated that temperatures exceeding 40 °C induce proton transport disorders, and the presence of crystalline vascularization in the mitochondria, mitochondrial swelling, and the formation of dense vesicles have been shown to adversely affect mitochondrial functionality [21–23]. The predominant disturbance is the membrane function, which in turn leads to mitochondrial depolarization and the release of reactive oxygen species (ROS), contributing to damage to cell Deoxyribonucleic Acid (DNA) [24, 25]. Furthermore, the release of the apoptotic factor cytochrome C from these structures serves to indirectly stimulate the process of apoptosis in target cells [26]. The process of DNA damage can transpire via multiple mechanisms. HT exerts a substantial effect on direct damage induced by RT. Additionally, HT restricts the capacity of cancer cells to repair the damage caused by RTH. The underlying mechanism of DNA damage induced by RT primarily involves the process of radiolysis of water molecules, which subsequently leads to the generation of ROS [27, 28]. These ROS, in turn, trigger a series of reactions that culminate in DNA oxidation [29]. However, it should be noted that direct ionization also plays a role in the overall process, albeit to a lesser extent. It is noteworthy that heat alone has been observed to elicit analogous effects, as previously documented. Specifically, elevated temperatures have been shown to augment DNA damage by fostering the generation of ROS. Additionally, it has been observed to influence DNA replication disorders and induce double-strand breaks (DSBs) by impeding the activity of DNA polymerase [16]. Histone proteins play a role in the process of heat-induced DNA damage by modulating the activation of the Ataxia Telangiectasia Mutated (ATM) protein [30]. Phosphorylated H2A histone family member X (γH2AX) foci formation is another consequence of heat-induced DNA damage, and it plays a role in the process of DSBs formation [30, 31]. In addition, heat can also directly contribute to the phosphorylation and activation of γH2AX [32]. The activation and formation cycle of these proteins is further supported by the activation of cellular pathway checkpoints (G1, G2, M), which results directly from the arrest of the cycle caused by DNA damage [31].

**Fig. 1:**
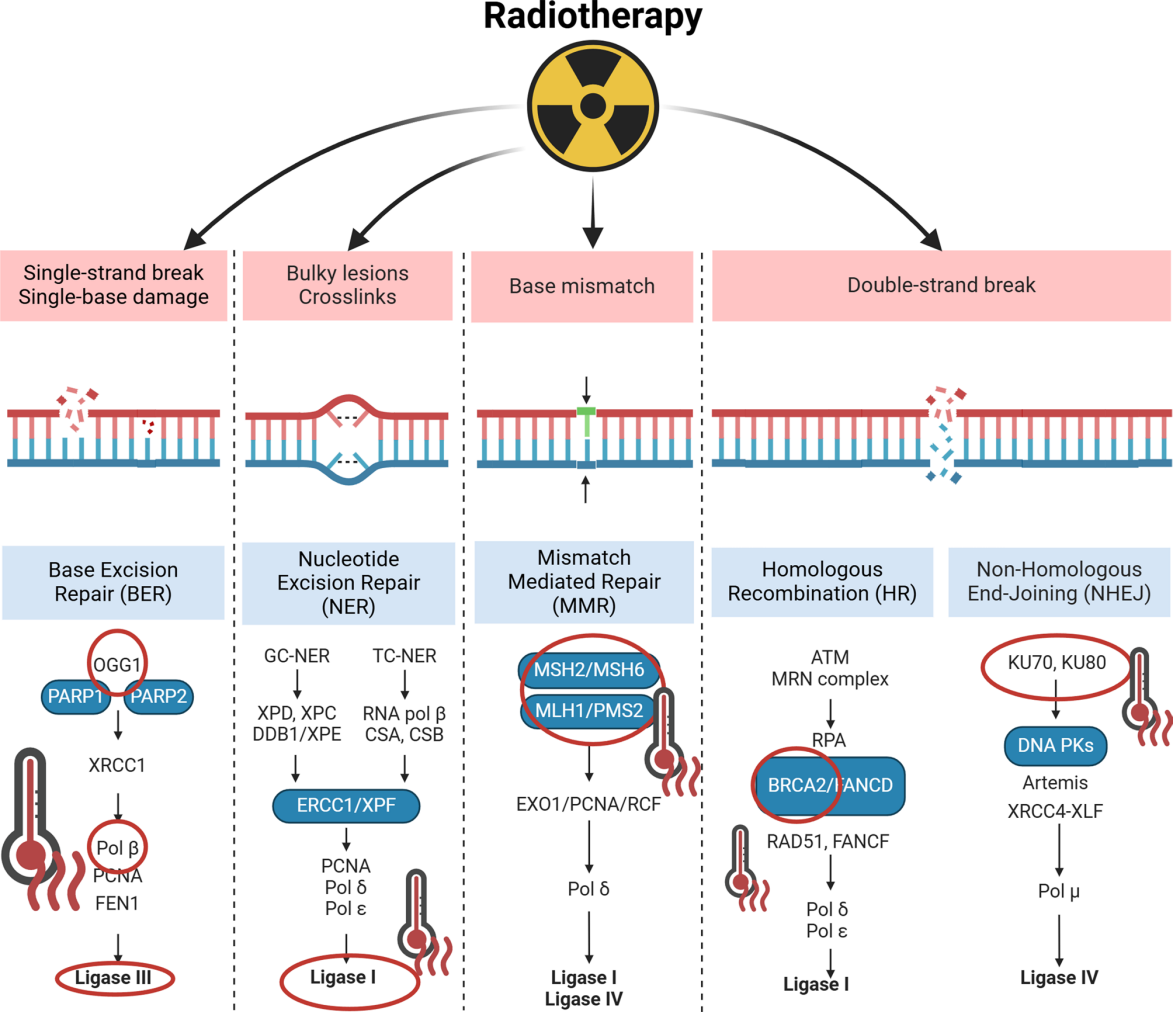
Potential mechanisms of HT in blocking DNA repair. HT has been observed to affect the BER, NER, and MMR pathways, in addition to NHEJ and HR. The activity of polymerases and ligases is blocked. HT inactivates OGG1 in the BER pathway. In the NER pathway, HT contributes to the formation of helix breaks and blocks the action of ligases. In the MMR pathway, the action of MMR factors hMLH1 and hMSH2 is inactivated. In the NHEJ pathway, KU and DNA binding is reduced under the influence of heat resulting in depletion of the activity of the DNA-PK complex. * ATM-Ataxia telangiectasia-mutated Kinase; BER- Base excision Repair; BRCA- BReast CAncer protein; CSA/CSB- cockayne syndrome protein A/B; DNA- Deoxyribonucleic Acid; DDB1- DNA damage binding protein; ERCC1- Excision Repair Cross Complementation Group 1; EXO1- exonuclease 1; FANCD/F- The Fanconi Anemia complementation Group; FEN1- Flap Endonuclease; GC-NER- Global Genome Nucleotide Excision Repair; Hr- homologous recombination; HT-hyperthermia; MLH1- mutL homolog 1; MMR- mismatch repair; MSH2/6- MutS homolog 2/6; NHEJ- non-homologous end joining; NER- nucleotide excision repair; OGG1- 8-oxoguanine DNA glycosylase; PARP1/2- poly (ADP-ribose) polymerase 1/2; PCNA- DNA polymerase processivity factor proliferating cell nuclear antigen; PK-DNA-dependent protein kinase; Pol- Polymerase; RAD51- Recombination Protein A; RCF- Replication Factor C; RNA-Ribonucleic Acid; RPA- Human Replication Protein A; TC-NER- transcription-coupled nucleotide excision repair; XPD/E/F- Xeroderma Pigmentosum Protein D/E/F; XRCC4- X-ray-complementing Chinese hamster gene 4

A pivotal synergistic effect of heat on RT is the inhibition of DNA damage repair (Fig. 1). The fundamental repair pathways influenced by HT encompass excision repair, comprising base excision repair (BER), nucleotide excision repair (NER), and mismatch repair (MMR), along with non-homologous end joining (NHEJ) and homologous recombination (HR). All of these pathways are involved in the repair of single-strand breaks (SSBs) and double-strand breaks (DSBs) induced by RT [33]. BER is predominantly hindered by temperatures in excess of 43 °C, as evidenced by studies demonstrating a substantial decline in BER in irradiated cells following the application of additional HT [34]. This phenomenon is primarily attributed to the thermal inhibition of DNA polymerase and its accompanying DNA glycosylases [35–37]. It has been demonstrated that at temperatures greater than 41 °C, and to a degree dependent on the further increase in temperature and time of HT application, there is a significant decrease in activity, especially polymerase beta [38]. Moreover, HT inactivates 8-oxoguanine DNA glycosylase (OGG1) by mobilizing its nuclear export and inducing its proteasome-mediated degradation, thereby impeding the recognition and removal of damaged DNA bases [39]. While the body of research on the involvement of NER is less extensive, the findings on the impact of HT, when added to CHT, are noteworthy. Specifically, the study observed that HT-induced ruptures in cross-links, which are crucial for the repair process, influenced the sensitivity of cells to treatment [40, 41]. In turn, the MMR mechanism is associated with the involvement of heat shock proteins (HSPs), which are induced by HT [25]. It has been demonstrated that MMR factors mutL homolog 1 (hMLH1) and MutS homolog 2 (hMSH2) are translocated from the nucleus into the cytoplasm in response to 41–42 °C heat shock, thereby disturbing the repair mechanism [42]. NHEJ, a predominant pathway that facilitates the restoration of DSBs in mammalian cells, functions independently of homologs or templates [43]. However, the role of HT in disrupting this mechanism remains to be fully elucidated, with the existing studies yielding contradictory results. Some studies that confirmed the influence of HT showed that it can affect the early stages of the NHEJ process by inactivating DNA binding by KU under the influence of heat and reducing the activity of the DNA-PK complex [44, 45]. This, in turn, correlated with the degree of radiosensitization at temperatures of 44–45 °C, as well as inducing aggregation of Ku factor in the cell nucleus [46]. Furthermore, the study demonstrated that reversible repression of DNA-PK activity by HT at 44 °C, and a significant decrease in the levels of KU70 and KU80 proteins was reported [47]. This resulted in the prevention of DNA binding and the processing of strand ends. The HR mechanism, which is active exclusively during the S and G2 phases of the cell cycle, is contingent on the presence of BRCA proteins [48]. It has been demonstrated that these proteins are a primary target of heat. Current studies have shown that temperatures above 41 °C lead to degradation of BReast CAncer gene 2 (BRCA2) and, to a lesser extent, BReast CAncer gene 1 (BRCA1) [49–51]. Furthermore, HT has been demonstrated to influence additional elements of the HR process, predominantly contributing to template blocking, homologous connections, and the inhibition of replication progression [52–54].

The majority of cellular structures are protein-based, and elevated temperatures directly contribute to the weakening or complete destruction of their protein skeleton. Among the various effects of HT on cancer cells, its impact on the structure of collagen fibers has emerged as a notable area of interest [55]. The migration and infiltration of the environment by cancer cells are significantly affected by collagen fibers [56]. Disturbances in their structure have been observed to restrict the spread of cancer and reduce its growth rate [57]. Furthermore, the integrity of the entire cellular cytoskeleton may be compromised, manifesting as intracellular shifts in microtubule positioning or the accumulation of actin filaments [58]. As previously stated, HSPs play a significant role in cell response to heat stress. Physiologically, these proteins are responsible for protecting cells from heat, causing refolding of proteins, preventing irreversible protein aggregation, repairing the centrosome and cytoskeleton, and cell death [59, 60]. This phenomenon, known as thermotolerance, is characterized by a temporary resistance of cells to heat and has a close correlation with clinical planning and the application of HT [61]. In addition to their direct impact on cell physiology, HSPs trigger molecular and immunological changes, thereby influencing the efficacy of HT in cancer treatment. The release of HMGB1 and HSP70 affects the tumor microenvironment (TME) through the activation of dendritic cells (DCs), increased presentation of antigens on T cells, and increased expression of MHC-I molecules, thus stimulating the immune system [62–66]. This topic will be discussed in more detail below.

**Fig. 2:**
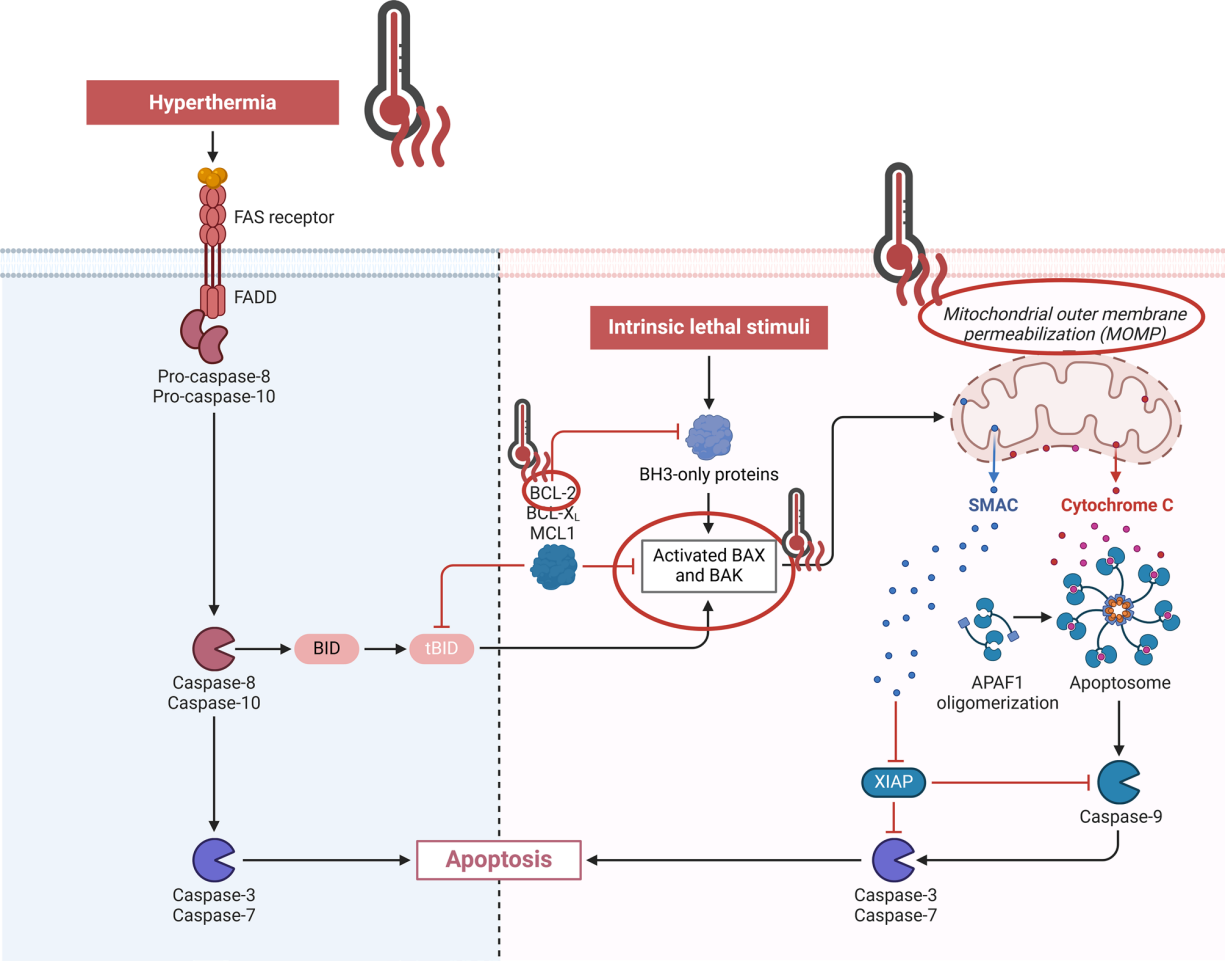
The impact of HT on apoptosis via the extrinsic (left)/intrinsic (right) pathway. HT may act as a direct catalyst, activating the extrinsic pathway through interactions with the death receptor FAS, thereby initiating the caspase cascade. In the intrinsic pathway, HT has been observed to induce the expression of proapoptotic genes while concomitantly repressing the expression of antiapoptotic genes. It has also been demonstrated to participate in the activation of caspase 2. Indirectly, it contributes to apoptosis by disturbing the membrane transport in mitochondria and depolarizing their membrane with the leakage of apoptosis-activating factors. *APAF1- Apoptotic Protease Activating Factor; BAK- Bcl-2 antagonist killer 1 protein; BAX- Bcl-2-associated X protein; BCL-2- B-cell lymphoma 2 protein; BH3- Bcl-2 homology domain 3.; BID- BH3 interacting-domain death agonist; FADD- Fas-associated death domain protein; MCL1-myeloid cell leukemia 1 protein; SMAC- mitochondriaderived activator of caspases; XIAP- X-linked inhibitor of apoptosis

HT has been demonstrated to induce cell death by disrupting a variety of physiological processes, some of which have previously been discussed. This cell death can occur via extrinsic/intrinsic apoptosis or mitotic catastrophe [67–70]. Mitotic catastrophe pertains to cells that are incapable of completing mitosis due to defects in the mitotic apparatus, DNA damage, and mitotic checkpoint errors [71]. Intrinsic apoptosis can be triggered by various intracellular signals, while extrinsic apoptosis is dependent on extracellular signals that lead to the activation of intracellular apoptotic pathways (Fig. 2.) [72]. HT has been demonstrated to influence the expression of pro-apoptotic genes. It has been observed that the application of elevated temperatures is associated with the upregulation of pro-apoptotic *Bax* and *FasL* genes, and the moderate expression or decrease of the anti-apoptotic *Bcl-2* gene [70, 73]. In the subsequent phases of the cellular pathways of these pro-apoptotic genes, HT has been demonstrated to stimulate the activation of caspase-2, which forms complexes with other signaling proteins, resulting in the release of cytochrome c from the mitochondria [74]. Subsequent complex formation activates the caspase pathway, ultimately leading to apoptosis [75, 76]. The extrinsic pathway of apoptosis can be stimulated by HT via increased expression of death receptors, as demonstrated in chordoma, where the combination of RT and HT resulted in increased apoptosis and expression of the FAS protein, which is a death receptor [77]. In addition, HT has been shown to inhibit the AKT signaling pathway, which is a survival cell signal [78]. As previously mentioned, HT exerts a substantial influence on the replication process in cells, leading to replication disorders, mitosis, chromosome aberrations, genome instability, and cell death by mitotic catastrophe [79].

The relationship between HT and tumor oxygenation is a complex process [80]. It is known that hypoxia in the TME leads to radioresistance [81, 82]. This phenomenon can be attributed to the fact that rapidly growing tumors experience an escalating demand for oxygen, resulting in the release of numerous factors that stimulate angiogenesis. However, this process is often conducted in an ineffective manner. The resultant vessels are dilated and heterogeneously distributed, failing to meet the tumor’s demand for oxygen, thereby creating a highly hypoxic TME. Hypoxia is a critical factor in radioresistance, as the underlying mechanism of cell damage by RT involves the formation of ROS, which requires sufficient oxygen. The interpretation of data concerning the effect of HT on the increase in tumor oxygenation subjected to RT remains challenging and is an area that requires further research. On one hand, mild HT has been shown to lead to vasodilation and increased oxygen delivery to cells [83]. On the other hand, elevated temperatures have been observed to increase cellular oxygen consumption. Furthermore, the presence of a well-vascularized network of tumor vessels in certain tumors can render them resilient to the effects of HT, potentially leading to the opposite outcome of destroying these vessels [84–86].

### Immunological effects of hyperthermia

**Fig. 3:**
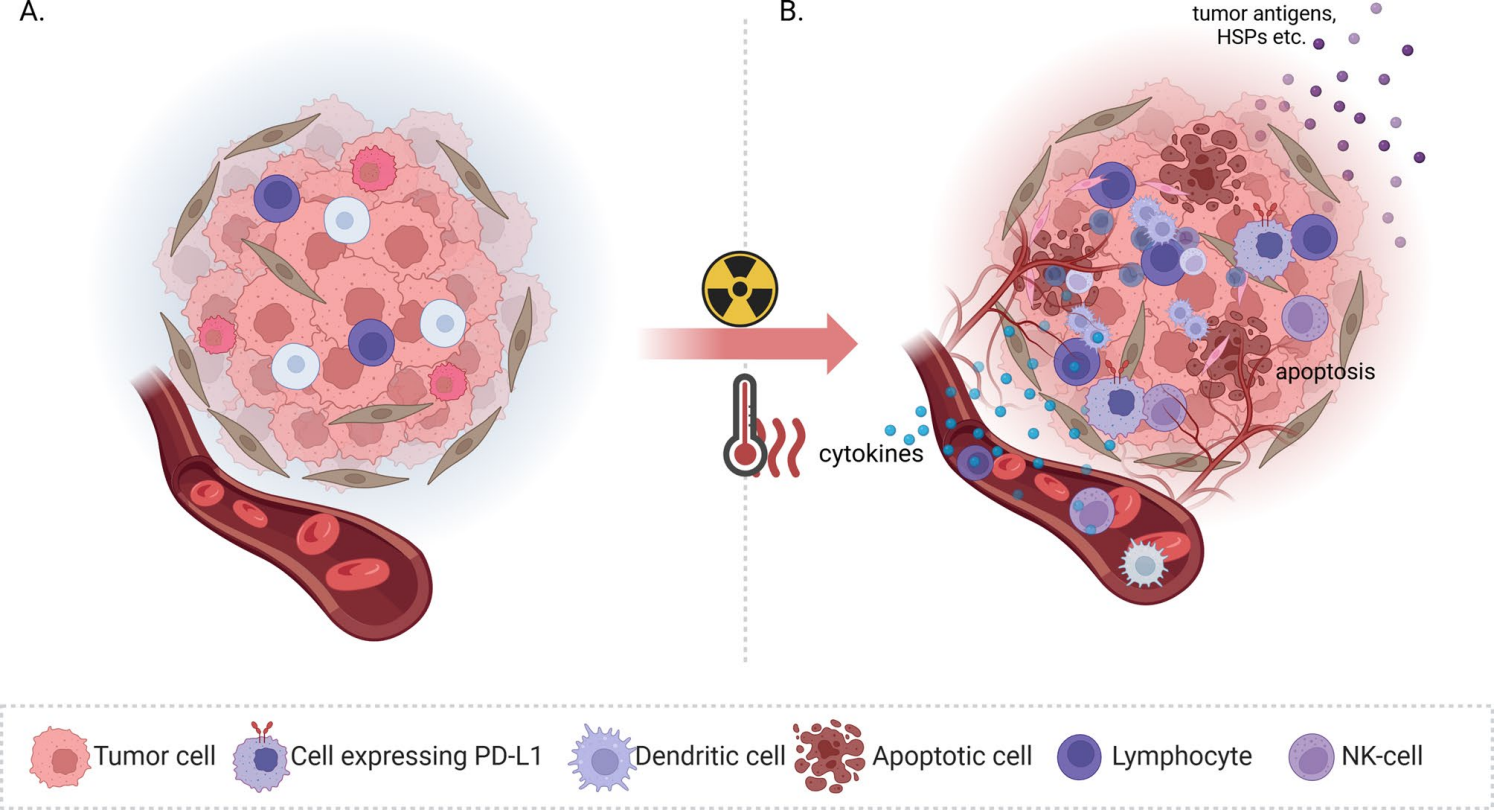
The effect of hyperthermia (HT) on the tumor’s immune microenvironment (TME). HT primarily causes increased perfusion and, consequently, oxygenation of the tumor. This phenomenon results in an augmented influx of immune cells into the TME. Furthermore, the process of apoptosis leads to an augmented release of tumor antigens and heat shock proteins (HSPs), which, in turn, results in an increased availability of these antigens for antigen-presenting cells (APCs). Consequently, the tumor microenvironment becomes more immunogenic, and the TH1/TH2 ratio increases. The release of various proinflammatory cytokines serves to further stimulate leukocyte activity. The expression of immune checkpoints also increases. In summary, HT can synergistically affect radiotherapy on the transformation of immunologically silent tumors (Fig. 3A) into more immunogenic tumors (Fig. 3B). * NK-cells- Natural Killer cells; PD-L1- Programmed Cell Death Ligand 1

The immunosuppressive properties of RT have been recognized for years; however, recent reports of its immunostimulating effect have become increasingly significant [87–89]. This is particularly relevant in the current era of ITH, where clinical studies have demonstrated the benefits of integrating RT into systemic therapy [90, 91]. Consequently, there is a compelling rationale for ongoing research endeavors aimed at integrating HT and other therapeutic modalities to augment this effect. HT itself has immunomodulating properties, especially within the temperature range characteristic of mild HT. As previously mentioned, HT leads to enhanced perfusion within the tumor, thereby increasing the infiltration of immune cells. Disturbances in DNA repair, which result in apoptosis, consequently render a greater proportion of tumor antigens available for immune system cells to recognize and respond to. The present chapter will primarily focus on the TME and the alterations in its dynamics induced by HT (Fig. 3).

First, in the case of HT, there is increased recruitment of antigen-presenting cells (APCs), their increased maturation, proliferation, and antigen expression [92]. In addition, receptors involved in the presentation of specific tumor antigens, including MHC II/CD80/CD86, and CCR7, are upregulated, causing an increased immune response [93]. Furthermore, there is migration of APCs to lymph nodes and increased communication between them and CD8+ lymphocytes [94]. In addition to the recruitment of APCs, HT has been demonstrated to cause increased aggregation and activation of CD8+ lymphocytes. Studies have shown that peripheral blood CD8+/CD28+ T cells increased (*p* = 0.002), while the CD4+/CD25+/CD127+ Treg cells decreased after heat therapy (*p* = 0.012) [95]. Furthermore, HT may have an impact on the shaping of T lymphocytes. It has been shown that HT leads to increased differentiation of CD4+ T lymphocytes towards Th1 and Th17 lymphocytes, while the percentage of developing immunosuppressive Treg lymphocytes is reduced [96, 97]. This differentiation may be a direct effect of heat therapy or an indirect effect of HT on the increased secretion of chemokine (CXCL10) and interleukin (IL-6) from M1-type macrophages, which promoted the differentiation of CD4 T cells towards Th1 [98]. Thanks to all these actions in the TME, the Th1/Th2 ratio increases and an immunologically active environment is created. HT has also been observed to stimulate other cells of the immune system, increasing the expression of MHC class II molecules on the surface of B cells, and influencing activation [99]. A notable effect that emerges from the combination of HT and RT is their ability to enhance the immunogenicity of cancer stem cells (CSCs), which often evade standard treatment methods under conventional conditions and are associated with relapses and cancer spread [78, 100]. Moreover, thermal therapy has been shown to enhance Th1 cytokines (IL-2, IL-12, IFN-γ, and TNF-α), thereby activating Th1 responses, and reducing Th2 cytokines (IL-4, IL-5, IL-13), thus suppressing Th2 responses, culminating in effective anti-tumor effects [101].

Immune checkpoints function as modulators of immune responses, with a role in the control of cancer progression [102]. The blockade of these molecules has been demonstrated to activate the immune system and effectively target cancer cells [103]. This principle underlies the current utilization of ITH. HT has been observed to enhance the expression of immune checkpoints on cells, and its combination with RT and ITH has been shown to have a positive effect [104]. In a study involving the use of RT with HT on breast cancer cells, including triple negative lines, a significant increase in PD-L1 expression was observed. However, this effect was not observed with HT alone [105]. Another study demonstrated an increase in PD-L1, PD-L2, HVEM, and Gal-9 expression after the use of HT alone, but this effect was intensified after the addition of R [106]. A similar outcome was observed in a study conducted on glioblastoma lines, wherein the highest increase in immune checkpoint expression was recorded at 41 °C [107].

### Achieving a biological effect

At present, the effect of HT, whether administered as a monotherapy or in combination with RT, on cancer cells is better understood. However, some effects have yet to be elucidated. The majority of the biological rationale for the utilization of HT originates from preclinical studies, which do not invariably translate directly into the outcomes observed in clinical trials [108]. The disparities can be addressed through a comprehensive analysis of the preceding chapters’ data. Three mechanisms, arising from molecular and immunological foundations, persist as the primary challenges associated with the combination of RT and HT: thermotolerance, radioresistance, and the abscopal effect. As previously mentioned, thermotolerance plays a pivotal role in resistance to heat, and tumor thermotolerance is the result of molecular mechanisms that involve the repair of structures, among others, by HSPs [109, 110]. Conversely, vessel thermotolerance pertains to the capacity of vessels to augment perfusion within the tumor, thereby facilitating the delivery of ROS, which in turn amplifies the efficacy of RT. It has been demonstrated that the tumor exhibits a markedly increased blood flow response to a second HT exposure in comparison to a single thermal dose, even at temperatures that would typically result in vascular damage [111, 112]. Consequently, effective intervals of HT administration in combination with RT are necessary, as in clinical practice, repeated HT treatments every 2–3 days may prove more beneficial than single applications [81, 113–115]. Radioresistance resulting from tumor hypoxia can be countered by increasing perfusion and oxygenation. Additionally, tumors originating from different tissues may exhibit varying vascular densities [116]. Furthermore, a high level of necrosis in the tumor and an extremely acidic environment significantly impede the use of RT with HT and overcoming radioresistance. In the context of heightened immune system stimulation by RT and HT, the abscopal effect has the potential to extend beyond the local treatment effect. The latter is defined as the regression of non-irradiated lesions distant from the treated lesions [117, 118]. However, the occurrence of this effect remains infrequent, and its biological basis remains to be elucidated. Consequently, elucidating these three mechanisms and adapting the clinical application of combined treatment to them may facilitate the optimization of the procedure.

## HT technique details

### Methods of hyperthermia

HT can be delivered through three primary clinical methods, each tailored to the tumor’s location, depth, and stage: local, regional, and whole-body HT [119]. These approaches target localized tumors, advanced or deep-seated malignancies, and disseminated cancers, respectively [120]. The energy sources used to induce HT include: microwaves (with wavelengths ranging from 433 to 2450 MHz), radiofrequency (ranging from 100 kHz to 150 MHz), ultrasound, hot water perfusion (using tubes or blankets), resistive wire implants, ferromagnetic seeds, magnetic nanoparticles, and infrared radiators [120, 121]. Each method is selected based on the specific clinical scenario, ensuring optimal heat delivery to the target tissue while minimizing damage to surrounding healthy structures. Thermometry in tumors is inherently invasive, as it requires direct temperature measurements, which can be associated with certain complications [5, 122]. To address these challenges, ongoing research is exploring the use of magnetic resonance imaging (MRI) as a non-invasive thermometry tool [123]. This advancement could revolutionize HT delivery by enabling real-time, accurate temperature monitoring without the need for invasive procedures. Furthermore, hyperthermia treatment planning (HTP) with the aid of specialized power and temperature distribution simulations is beneficial. Treatment planning systems for HT have advanced in sophistication in recent years and are now commercially accessible for clinical usage. By enabling the visualization and optimization of thermal dosage patterns, these devices help to improve the safety and quality of treatments. Recent quality assurance guidelines for HT promote HTP, which is being included in clinical workflows [124]. The use of HTP allows for more precise, individualized treatments and supports the safe escalation of thermal dose to the tumor while minimizing risks to healthy tissues [125, 126]. HTP contributes to clinical decision-making by enabling the assessment of a particular treatment plan or the comparison between different therapeutic options. In superficial hyperthermia, this usually involves examining the quality of heating. For locoregional hyperthermia performed with phased array systems, HTP is useful in analyzing how modifications in phase and amplitude affect the anticipated heating patterns. Moreover, HTP is tailored to diverse clinical contexts and to the hyperthermia devices available within the facility [127]. Sigma HyperPlan is one of the most used commercial HTP systems, created especially for deep HT with the BSD2000 system. By simulating field and temperature distributions using finite element methods, Sigma HyperPlan enables patient-specific modeling and heating strategy optimization [124–126]. SEMCAD X and COMSOL Multiphysics are two other noteworthy tools [125, 126]. These versatile platforms may simulate particular absorption rates, temperature distributions, and phase-amplitude optimization for a range of applicator systems.

### Local hyperthermia

Local HT is designed for treating tumors that are either superficial (located within a few centimeters of the skin surface) or accessible within body cavities, such as the rectum or esophagus [1]. Typically, local HT is most effective for tumors with a maximum dimension of ≤ 3 cm for superficial lesions, and up to 5–6 cm for deeper or intracavitary lesions, provided they are within the penetration depth of the chosen energy source [125, 127]. The penetration depth depends on the applicator and energy type, with microwaves and radiofrequency waves generally reaching 3–5 cm below the skin surface [126]. This method employs superficial, interstitial, intraluminal, or intracavitary applicators, with heat delivery primarily achieved using microwaves, radiofrequency waves, or ultrasound [128]. Local HT techniques allow precise heating of cancerous areas while sparing surrounding normal tissues. For superficial tumors (located in or just beneath the skin), specialized applicators or antennas (e.g., waveguides, spiral, current sheet, or compact designs) are placed directly on or near the target area. To minimize side effects, such as skin blistering or burns, cooling systems like water boluses are often used during treatment [129]. These systems maintain the skin temperature at approximately 37 °C by using cooling devices (such as water boluses), which protect superficial tissues while allowing adequate heating of the tumor at depth [1]. Interstitial HT is another technique, suitable for tumors treatable with brachytherapy, including head and neck cancers, prostate cancer, breast cancer, and brain malignancies. This method involves inserting tiny microwave antennas, radiofrequency electrodes, ultrasound transducers, heat sources, or laser fibers into the tumor tissue using brachytherapy applicators. Heating can be applied for up to 2 hours before or immediately after radiation [130]. A promising advancement in local HT is the use of magnetic nanoparticles. In this approach, iron oxide nanoparticles are injected directly into the tumor and heated using an external magnetic field [131]. This method enables precise heating of deep-seated tumors, such as recurrent glioblastoma in the skull or pelvic tumors like prostate and cervical carcinomas, offering a minimally invasive alternative for challenging locations [132].

### Regional hyperthermia

Regional HT targets larger areas, such as the pelvis, abdomen, or limbs, and is commonly used for advanced or deep-seated tumors. Techniques include deep tissue heating with external applicators (e.g., phased-array systems), regional perfusion, and continuous hyperthermic peritoneal perfusion (CHPP) or hyperthermic intraperitoneal chemotherapy (HIPEC) for abdominal malignancies [1, 133].

External applicators, typically consisting of coherent arrays of dipole antenna pairs arranged in a ring around the patient, are used to deliver microwave or radiofrequency energy to deep-seated tumors [134]. These systems can heat targeted anatomical regions to 41–42 °C, with the temperature limited by power deposition in surrounding tissues [134]. Other deep-heating methods include capacitive and inductive techniques, both utilizing radiofrequency sources. Recent advancements aim to develop planning and monitoring systems for deep regional HT, often leveraging MRI for non-invasive temperature and perfusion monitoring [126].

Regional perfusion is a technique used to treat malignancies in the limbs (e.g., melanoma) or organs (e.g., liver, lung). It involves bypassing a major artery and vein of the affected limb or organ to heat the blood extracorporeally before returning it to the body [135].

CHPP and HIPEC are used to treat cancers that have spread within the abdominal cavity, such as peritoneal carcinomatosis, multifocal ovarian or colorectal metastases, mesothelioma, and stomach cancer [136, 137]. The treatment strategy involves surgical resection of the primary tumor and cytoreduction of peritoneal and, in some cases, liver metastases. During the procedure, warmed fluid (41.0–42.5°C), with or without chemotherapeutic agents, is circulated through the peritoneal cavity to target residual cancer cells. HIPEC, in particular, combines the cytotoxic effects of heat with chemotherapy, enhancing treatment efficacy [138].

### Whole-body hyperthermia

Whole-body hyperthermia (WBH) may be a therapeutic option for patients with metastatic diseases such as melanoma, soft tissue sarcoma, or ovarian cancer [139]. The goal of WBH is to destroy or sensitize disseminated cancer cells by raising the body’s core temperature. This is achieved using thermal chambers, hot water blankets, or infrared radiators, combined with measures to prevent heat loss and maintain electrolyte balance [119]. WBH is administered in two main forms. Firstly, extreme WBH, where patients are heated to approximately 42.0 °C for 60 minutes under general anesthesia or deep sedation. Secondly, moderate WBH, where patients are heated to 39.5–41.0 °C for 3 to 4 hours, typically requiring sedation. Common side effects include transient diarrhea, nausea, and vomiting [140].

### Hyperthermia limitations and perspectives

Despite the significant potential of HT in oncological treatment, and its early stage of development and future improvements, there are inherent limitations that affect current clinical management. A substantial impediment pertains to the emergence of deleterious high-temperature regions (hotspots) within normal tissue, which can result in discomfort or burns [127]. Treatment systems planning has been demonstrated to facilitate the prediction and mitigation of these symptoms. It is imperative to achieve effective cooling of the skin and normal tissue, particularly in cases of superficial and regional HT [126]. Water boluses and other cooling systems are standard, but their effectiveness can be limited by patient anatomy or device design. The variability in anatomy, tissue perfusion, and tumor location necessitates individualized planning. The advent of modern HTP systems has rendered patient-specific modeling increasingly feasible. Furthermore, patient anatomy (e.g., obesity) may render certain HT techniques contraindicated. Temperature monitoring is usually performed with invasive probes—such as thermistors, fiber optics, or thermocouples—placed in or near the tumor and oriented approximately perpendicular (≈90°) to the electromagnetic field to reduce measurement errors [141]. Non-invasive methods, such as MRI thermometry, are currently undergoing development but have yet to be standardized [142].

In addition, recent studies have identified prospects for future research in the field of HT. For instance, WBH, despite its limited utilization in clinical settings, is undergoing development in the context of clinical trials. Preliminary findings indicate the possibility of synergistic effects with immune checkpoint inhibitors; however, further investigation is necessary to substantiate these observations [143]. The investigational focus at present is twofold: firstly, the potential of WBH in the context of metastatic disease, and secondly, its role as an adjunct to immunotherapy. Recent preclinical and early clinical studies suggest that combining WBH with immune checkpoint inhibitors may enhance anti-tumor immune responses and improve outcomes [143]. A number of clinical trials are currently underway to assess the safety and efficacy of this combination in various malignancies [144]. However, the extant evidence remains limited, and further large-scale studies are needed to establish its role.

## Clinical evidence for radiotherapy with hyperthermia

In order to assemble clinical evidence regarding the effectiveness of combining RT with HT, clinical trials in this area were retrieved and summarized per tumor entity. The electronic database of PubMed was systematically searched for articles published until February 2025. The main search strategy included a keyword search according to the following formulas: ((Hyperthermia[Title/Abstract]) OR (HT[Title/Abstract]) AND (Radiotherapy[Title/Abstract]) OR (RT[Title/Abstract]); and depending on the diagnosis: AND (Breast cancer[Title/Abstract]); (Cervical cancer[Title/Abstract]); (Bladder cancer[Title/Abstract]); (Prostate cancer[Title/Abstract]); (Prostate cancer[Title/Abstract]); (Sarcoma[Title/Abstract]); (Melanoma[Title/Abstract])). The main eligibility criteria included prospective clinical trials and meta-analyses; retrospective studies, case reports, narrative reviews, non-English articles, and duplicates were excluded. The search was supplemented by manually screening the reference lists of the articles that were selected based on the search. Titles and abstracts were screened independently by two authors; disagreements were resolved through assessment by two reviewers (P.C./A.B.). The results were assessed in terms of meeting the inclusion criteria, study quality and availability of endpoint reporting such as local control, response rate, PFS, OS and toxicity. In the end, 941 records were retrieved; 877 were excluded (wrong design/insufficient data/duplicates); 57 prospective trials and 7 meta-analyses were included.

### Breast cancer

The majority of studies on the use of RT in combination with HT in breast cancer primarily refer to its application in cases of inoperable recurrence of the neoplastic process, recently, a significant focus is placed on HT use in locally advanced primary tumors. (Table 1). Consequently, HT was employed in the National Comprehensive Cancer Network (NCCN) guidelines for the treatment of recurrent breast cancer [12]. Given that RT is an integral component of the treatment of the primary tumor in cases of re-irradiation, HT may offer additional benefits, particularly in scenarios where reduced doses of RT are employed [189, 190]. One of the first studies on the combination of RT and HT in the treatment of recurrent breast cancer included 30 patients with recurrent or metastatic disease who had previously undergone multimodality treatment, including surgery, radiation therapy, CHT, and hormonal treatment. This phase II study demonstrated that, despite the implementation of low-dose re-irradiation augmented with HT, substantial outcomes could be attained, as evidenced by the achievement of complete rate (CR) in 57% of patients and the duration of local control ranging from 6 to 32 months. The progression of the tumor process at the irradiated site was observed in a low number of patients. Despite the intensive local treatment, the rate of adverse events remained low [151]. Subsequent studies demonstrated that the incorporation of HT led to a substantial reduction in the required RT doses while maintaining a robust local effect [191]. Furthermore, clinical studies sought to ascertain the correlation between the parameters of the HT utilized and the efficacy of treatment for recurrent breast cancer. MaxTDmin, defined as the maximum of the lowest thermal dose recorded at any measurement point during a treatment, was subjected to analysis. A summary of four clinical trials demonstrated that the local CR rate was 74% for MaxTDmin > 6 min and 47% for MaxTDmin ≤ 6 min (*p* = 0.009) [192]. Also, triple therapy with RT, HT, and CHT was found to be effective and safe for this indication [147, 148]. A study on primary advanced breast cancer demonstrated contradictory results. A phase III study compared RT to RT+HT in superficial tumors, including 68 patients with breast cancer. However, HT did not demonstrate superiority in terms of objective response rate [(ORR), sum of partial responses (PR) and complete responses (CR)] or local control (LC) [150]. As this is one of the older reports, it is difficult to draw conclusions from it regarding the combination of these methods in a modern treatment protocol. A subsequent study pooled the results from five randomized trials, indicating a significantly better CR rate (RT+HT: 59%, RT alone: 41%, *p* < 0.001) but no survival benefit [149]. Notably, 50% of the patients included had already been diagnosed with metastatic disease at the time of the study procedures. More recent reports have compared RT with HT to RT alone, including patients with primary breast cancer who had never been previously irradiated. The analysis revealed that only 4 out of 57 patients experienced recurrence within the irradiated area [146]. Last year’s analysis of HT in advanced breast cancer showed the most promising results as the 5-year actuarial local failure rate was 57% in the whole group 68% in the RT+HT group and 50% in RT alone (*p* = 0.04) [145]. A meta-analysis of studies on the use of RT+HT included studies on locally recurrent breast cancers. A comprehensive review of the extant literature was conducted, encompassing a total of thirty-four scientific studies. This analysis included five randomized clinical trials, wherein patients were administered a median of seven HT sessions, resulting in an average temperature of 42.5 °C. The mean RT dose was determined to be 38.2 Gy, with a range of 26 to 60 Gy. A substantial advantage of HT was demonstrated, and CR of 60.2% was achieved with RT + HT versus 38.1% with RT alone (risk ratio 1.57, 95% CI 1.25–1.96, *p* < 0.0001) [152].

**Table 1:** Summary of the findings from clinical trials conducted on the combination of radiotherapy and hyperthermia. 5-FU- 5-fluorouracil, AEs- adverse events, BR- brachytherapy, CR- complete response, d- day, DFS- disease-free survival, DSS- disease-specific survival, EBRT- external beam radiation, EFS- event-free survival, FIGO-International Federation of Gynecology and Obstetrics, HNC- head and neck cancer, ht- hyperthermia, LC- local control, LPFS- local progression-free survival, LRC- loco-regional control, *m*- median, NA- non applicable, OR- odd ratio, ORR- objective response rate, OS- overall survival, pCR- pathological complete response, PFS- progression-free survival, PR- partial response, PSA-Prostate-specific antigen, RCT- randomized clinical trial, RFS- recurrence-free survival, SD- stable disease

Author, year	Study type	Phase	Number of patients enrolled	Diagnosis details	Radiotherapy details				Hyperthermia details	Additional treatment details	Outcomes	Reference
					Fraction dose (Gy)		Total dose (Gy)					
Overgaard et al. 2024	RCT	III	142	T2-4 advanced breast cancer	2		65–70		43 °C, 60 minutes, weekly, 6 sessions	NA	5-year local failure rate RT alone 68% vs RT + HT 50%, *p* = 0.04	[145]
Varma et al. 2012	Prospective within-patient comparative study	III	59	Locally advanced breast cancer (T3, T4, or more than three involved nodes or local recurrence)	1.8–2		46–50, boost to the total dose 66		41 °C, 60 minutes, weekly, 4 sessions	NA	30% recurrence rate	[146]
Kouloulias et al. 2002	Single-arm clinical trial	I/II	15	Recurrent breast cancer	1.8		30.6		NA	Liposomal doxorubicin 40–60 mg/m^2^ weekly	20% CR rate, measurable response in all patients	[147]
Feyerabend et al. 2001	Single-arm clinical trial	I/II	25	Recurrent breast cancer	1.2/2		Adjusted to the remaining tissue tolerance, 30–70		43 °C, 60 minutes, weekly	Epirubicin 20 mg/m^2^ and ifosfamide 1.5 g/m^2^ , weekly	ORR rate 80%, CR rate 44%	[148]
Vernon et al. 1996	Pooled RCT	III	307	Advanced breast cancer	2		60		43 °C, 60 minutes, weekly	NA	CR rate 59% in the RT+HT group vs. 41% in the RT group (*p* < 0.001)	[149]
Perez et al. 1991	RCT	III	65	Breast cancer	4		32		42.5 °C, 60 minutes, 2xweek, 6–8 sessions	NA	CR rate 62% in the RT+HT group vs. 40% in the RT group (*p* = 0.41)	[150]
Dragovic et al. 1989	Observational prospective single-arm study	II	30	Recurrent breast cancer	4		32		43 °C, 60 minutes, 2xweek, 6–8 sessions	NA	93% ORR rate	[151]
Datta et al. 2016	Meta-analysis	NA	2110	Recurrent breast cancer	1.8–4		38.2		42.5 °C, 7 sessions	NA	CR of 60.2% in RT + HT versus 38.1% in RT alone	[152]
Wang et al. 2020	RCT	NA	373	Cervical cancer FIGO stage IB (63.5%)- IVB	EBRT:1.8/2.0 BR:5		EBRT:50/50.4 BR:20/25		40.5 °C (39.5°C-41.5°C), 60 minutes, 2x week, 2–6 sessions	Cisplatin 30 mg/m^2^, d1-3; 5-fluorouracil 350 mg/m^2^, d1-5	5-years OS 81.9% in HT group vs.72.3%, *p* = 0.04	[153]
Lutgens et al. 2016	RCT	III	87 (terminated due to insufficient recruitment)	Cervical cancer FIGO stage IB–IIA (⩾4 cm)- IIB–IVA	EBRT: 2.0 BR:21,29,32		EBRT: 50 BR: 7, 4–7		60 minutes, weekly, up to 5 sessions	Cisplatin 40 mg/m^2^	Cumulative EFS 35 vs. 33%, *p* = 0.7	[154]
Harima et al. 2016	RCT	II	101	Cervical cancer FIGO stage IB–IIA (⩾4 cm)- IIB–IVA	EBRT:1.8/2.0 BR:5–6		EBRT:50/50.4 BR:20–30		40.1–44.6 °C, 60 minutes, weekly, 1–6 sessions	Cisplatin 30–40 mg/m^2^ weekly, 3–5 cycles	5-year OS 77.8% in HT group vs.64.8%, *p* = 0.141, CR rate 88.0% in HT group vs. 77.6%, *p* = 0.047)	[155]
Zolciak et al. 2013	RCT	III	205	Cervical cancer FIGO stage II–III	EBRT:1.8/2.0 BR*:7.5 *2–3 weeks after EBRT		EBRT: 45–50 BR*:30 2–3 weeks after EBRT		42.5 °C, 60 minutes, weekly, with BR, 4 sessions	Cisplatin 40 mg/m^2^, weekly with EBRT	3-year DFS 60% in HT group vs. 67%, *p* = 0.174	[156]
Zolciak et al. 2012	Prospective non-randomized	I	76	Cervical cancer FIGO stage I (8%)- III	EBRT:1.8/2.0 BR*:7.5 *2–3 weeks after EBRT		EBRT: 45–50 BR*:30 2–3 weeks after EBRT		42.5 °C, 60 minutes, weekly, with BR, 4 sessions	Cisplatin 40 mg/m^2^, weekly with EBRT	5-year DFS 65.8% in HT group vs. 73.6%	[157]
Franckena et al. 2008	RCT	III	114	Cervical cancer FIGO stage IIB (⩾4 cm)–IVA	EBRT: 2.0–1.8 BR: 6.0/8.5		EBRT:46–50.4 BR: 17/18		Max 42 °C, 90 minutes, weekly, up to 5 sessions	NA	12-year OS 37% in the HT group vs. 20%, *p* = 0.03	[158]
Sreenivasa et al. 2006	Single-arm clinical trial	II	32	Cervical cancer FIGO stage IIB (⩾4 cm)–IVA	EBRT: 1.8		EBRT: 45–50.4, boost up to 59.4–63		43 °C, 60 minutes, weekly, 1–5 sessions	Cisplatin 40 mg/m^2^, weekly	3-year OS and EFS 60%, 58%, respectively. R0 resection in 81% of pts	[159]
Westermann et al. 2005	Single-arm clinical trial	I/II	68	Cervical cancer FIGO stage IB (inoperable), IIB,IIIB, IVA	EBRT:1.8/2.0 BR: scheme varied depending on the location		EBRT:45–50.4 BR: scheme varied depending on the location		Exceeding 40 °C, 60 minutes, weekly, 4 sessions	Cisplatin 40 mg/m^2^, weekly	2-year DFS and OS 71.6% and 78.5%, respectively	[160]
Vasanthan et al. 2005	RCT	NA	110	Cervical cancer FIGO stage IIB-IVA	Parameters varied across study locations		Parameters varied across study locations		Parameters varied across study locations	NA	3-year OS 73.2%, no significant differences between groups	[161]
Tsuda et al. 2003	Prospective single-arm	I	15	Cervical cancer stage I-III	EBRT:1.8		EBRT:50.4		≥41 °C, 60 minutes, weekly, 4 sessions	Intra-arterial carboplatin 25 mg/m^2^	mOS 22.3 months (range 7–70 months), mPFS 8.9 months (range 2–55 months)	[162]
van der Zee & González 2002/van der Zee et al. 2000	RCT	NA	114	Cervical cancer FIGO stage IIB-IVA	EBRT: 1.8–2 BR:8.5		EBRT:46–50.4 BR:17, 20–30		42 °C, 60 minutes, weekly, 1–6 sessions	NA	3-year OS 51% in the HT group vs. 27%, *p* = 0.009	[163, 164]
Harima et al. 2001	RCT	NA	40	Cervical cancer FIGO stage IIIB	EBRT: 1.8 BR:7.5		EBRT:52.2 BR:30		41.8 ± 1.1 °C, 60 minutes, weekly, 3 sessions	NA	3-year OS 58.2% in the HT group vs. 48.1%, *p* = 0.3, 3-year RFS 79.7 in the HT group vs. 48.5, *p* = 0.048	[165]
Dinges et al.	Single-arm clinical trial	II	18	Cervical cancer TNM staging T2b-T4	EBRT: 1.8 BR:5		EBRT: 50.4 BR:20 if BR was not possible EBRT boost of 14.4 was given		≥41 °C, 90 minutes, weekly, 2 sessions	NA	2-year DSS 31.8%	[166]
Sharma et al. 1991	RCT	NA	50	Cervical cancer FIGO stage II-III	EBRT:2.25 BR:NA		EBRT:45 BR:35		42–43 °C, 30 minutes, weekly, 3 sessions	NA	Results completed per patient	[167]
Sharma et al. 1990	RCT	NA	50	Cervical cancer FIGO stage II-III	As in the previous line					NA	Evaluation of AEs	[168]
Yea et al. 2021	Meta-analysis	NA	536	Cervical cancer FIGO stage I-IV	EBRT: 1.8 BR: 4–5	EBRT: 50.4–52.2 BR: 20–30		Average 40.5/6 °C for 60 min		Cisplatin-based	Superior 5-year OS in HT group vs non- HT group (HR 0.67, 95% CI 0.47–0.96, *p* = 0.03).	[169]
Datta et al. 2019	Meta-analysis	NA	9894	Cervical cancer FIGO stage I-IV	EBRT:1.8–2.25 BR: various		EBRT: 30.6–64.8 BR: various		Various	Cisplatin-based	Superior LRC in HT group vs sole RT group, OR (2.61; 95% CI, 1.55–4.39; *p* < 0.001).	[170]
Datta et al. 2016	Meta-analysis	NA	1160	Cervical cancer FIGO stage I-IV	EBRT: 1.8–2.25 BR: 7.5–35		EBRT: 40–70 BR: various		40–43 °C, around 60 min	Cisplatin-based	Superior LRC in HT group vs RT group, OR 2.61 (95% CI: 1.55–4.39, *p* < 0.001).	[171]
Anscher et al. 1992	Single-arm clinical trial	I/II	12	T2b-T4 prostate cancer	EBRT:1.8–2		EBRT:65–70		≥42.5 °C, 60 minutes, once/twice a week	NA	3-year OS 88%, 3-year biochemical DFS 25%	[172]
Fosmire et al. 1993	Single-arm clinical trial	I	14	Prostate cancer C2–D1 (American Urological Society Stage)	EBRT:1.8–2		EBRT:67–70		≥42.5 °C, 30 minutes	NA	Evaluation of AEs	[173]
Algan et al. 2000	Single-arm clinical trial	I/II	26	Prostate cancer C2–D1 (American Urological Society Stage)	EBRT:2		EBRT:68		≥42.5 °C, 30 minutes, 1–2 sessions	NA	5-year OS 73%, 5-year biochemical DFS 35%	[174]
Deger et al. 2002	Single-arm clinical trial	II	57	T1c-T3 Prostate cancer	EBRT:1.8		EBRT:68.4		42–46 °C	NA	Initial PSA 11.6 ng/mL, after 3, 12, 24 months −2.4, 1.3 and 0.55 ng/mL respectively	[175]
Tilly et al. 2005	Single-arm clinical trial	I/II	22	T3N0M0 Prostate cancer	EBRT:1.8		EBRT:68.4		> 41.5 °C,weekly, 5–6 sessions	NA	6-year OS 95% in HT group vs 60% *p* < 0.05	[176]
Hurwitz et al. 2011	Single-arm long-term follow-up study	II	37	T2b-T3b N0M0 prostate cancer	EBRT:1.8–2		EBRT:66.6 (63.4–72)		Average 41.2 °C	NA	7-year OS 94%, 5-year biochemical DFS 61%	[177]
Beck et al. 2021	Single-arm (interim analysis) clinical trial	II	52	Biochemically recurrent prostate cancer	EBRT:2		EBRT:70		≥40 °C, 60 minutes, 10 sessions	NA	Initial PSA median 0.25 ng/mL (0.07–0.77), 3-month PSA median 0.085 ng/mL (0–5.31)	[178]
Spałek et al. 2021	Prospective single-arm clinical trail	II	30	Locally advanced marginally resectable or unresectable STS	EBRT*:3.25 *if unfitted for surgery +16		EBRT*:32.5 64		39–42 °C, 4 sessions* *if unfitted for surgery +2 sessions	NA	1-year LPFS 93%, 1-year PFS 88%	[9]
Maguire et al. 2001	Single-arm clinical trial	II	35	High-grade STS	EBRT:1.8–2		EBRT:50		2x week	NA	pCR in 52% of patients	[179]
Prosnitz et al. 1999	Single-arm clinical trial	II	97	Grade 2/3 resectable STS	EBRT:1.8–2		EBRT:50–50.4		42.5 °C, 60 minutes, weekly/2xweek	NA	10-year OS and RFS both 47%	[180]
Leopold et al.	Single-arm clinical trial	II	17	STS stage IIB-IVA	EBRT:1.8–2		EBRT:50–50.4		42 °C, 60 minutes, once a week vs. two times a week	NA	1 CR, 4 PR, 4 SD in HT 2xweek vs. 0 CR, 1 PR and 7 SD.	[181]
Overgaard et al. 1995	RCT	NA	70	Recurrent or metastatic melanoma	EBRT:8/9		EBRT:24/27		43 °C, 60 minutes	NA	2-year LC 46% in the HT group vs. 28%, *p* < 0.05	[182]
Zschaeck et al. 2021	Single-arm clinical trial	II	10	Previously treated head and neck squamous cell carcinomas	EBRT:1.7 +2.2 boost for macroscopic disease Re-irradiation:1.2		EBRT:54.4+70.4 Re-irradiation: 66		38.5–39.0 °C, 60 minutes, once a week	Cisplatin 30 mg/m^2^ Cisplatin 2cycles of 5 doses 20 mg/m^2^ during week 1 and 5 of RTH	Median time to local recurrence 10.5 months	[183]
Kang etal. 2013	RCT	NA	154	Nasopharyngeal carcinoma with nodal involvement	EBRT:2		EBRT:78		42.5–43.0 °C, 45 minutes, 2xweek, 3–14 sessions	Cisplatin 80 mg/m^2^	CR rates in HT and control group 81.6% and 62.8% (*p* < 0.05), 5-year OS 68.4% in HT group vs.50.0% *p* = 0.001	[184]
Hua et al. 2011	RCT	III	180	Nasopharyngeal carcinoma	EBRT:2		EBRT:70		42.5–43.0 °C, weekly	cisplatin (100 mg/m^2^, day 1) by 5-FU (1 g/m^2^/24 h for 4 days, days 1–4), 2–3 cycles	CR rates in HT and control group 95.6% and 81.1%, respectively *p* = 0.003, 5-year OS 78.2% in HT group vs.70.4% *p* = 0.14	[185]
Huilgol et al. 2010	RCT	NA	56	Locally advanced HNC	EBRT:2		EBRT:70		42.3 °C, 30 minutes, weekly	NA	CR rates in HT and control group 78.6% and 42.4% respectively *p* < 0.05	[186]
Serin et al. 1999	Single-arm pilot study	II	21	Recurrent HNC with nodal involvement	EBRT:2		EBRT:60–70 Non-metastatic nodes: 46		42.0 °C, 60 minutes, 2xweek, 3–8 sessions	Cisplatin 30 mg/m^2^, 2–6 cycles	1-year OS 39%	[187]
Valdagni et al. 1994	RCT	III	41	HNC stage IV	EBRT:2–2.5		EBRT:64–70		42.5 °C, 30 minutes, 2xweek	NA	5-year OS rates 53.3% in HT group vs 0% in control *p* = 0.02	[188]
Datta et al. 2015	Meta-analysis	NA	451	HNC stage I-IV	1.8–2.0		32–80		42.5 °C, 20–45 min, once/2x week	NA	Overall CR rate in HT group 62.5% vs 39.6% in RT alone group	[7]

### Cervical cancer

According to the current guidelines established by the European Society of Gynecological Oncology (ESGO) and the European Society for Radiotherapy and Oncology (ESTRO), HT constitutes an alternative treatment modality for cases of locally advanced cervical cancer. In circumstances where cisplatin is contraindicated, alternative treatments such as carboplatin or HT can be considered [11]. The efficacy of combining RT with HT and/or CHT has been demonstrated in several clinical trials. In general, greater treatment efficacy was demonstrated after adding HT to RT, and triple regimens combining RT, CHT, and HT were particularly effective. Notably, despite the heightened intensity of these treatment regimens, no substantial escalation in adverse events was observed. The results of these studies are summarized in Table 1 [153–168]. However, early research on this combination in cervical cancer was marked by a lack of uniformity concerning HT delivery, HT schedules, as well as RT treatment protocols, RT dose, and RT techniques. The majority of studies concentrated on the utilization of external-beam radiation (EBRT) and brachytherapy (BR). One of the studies incorporated intra-arterial carboplatin administration as a form of CHT. However, the patient population in these studies was predominantly homogeneous, with most studies including patients diagnosed with FIGO ≥ II or bulky type (FIGO IB ⩾4 cm). However, recent studies have expanded the patient population to comprise cases of the FIGO IB stage. In this study, 373 patients received standard radiochemotherapy, EBRT at a dose of 1.8 to 2.0 Gy to 50.4 Gy or 50 Gy with a cisplatin and 5-fluorouracil regimen. HT was administered at a temperature of 40.5 °C for 60 minutes, with each session repeated twice a week, totaling six fractions. In this trial, the 5-year overall survival (OS) rate in the RT+HT group (81.9%) was higher than that in the RT group (72.3%) (*p* = 0.04). No significant differences were observed in local recurrence-free survival (RFS). The stage of the disease was found to be a negative predictor of OS [153]. Furthermore, an analysis of HT parameters was conducted based on one of the clinical studies, which demonstrated that higher thermal dose parameters were associated with superior local RFS, disease-free survival, and ORR to treatment [193]. To systematize these results, a meta-analysis was conducted evaluating the addition of HT to chemoradiotherapy in locally advanced cervical cancer. A group of 536 patients was evaluated, and the results demonstrated that the patients receiving HT exhibited a significantly higher five-year OS rate compared to the patients who received chemoradiotherapy alone (HR 0.67, 95% CI 0.47–0.96, *p* = 0.03). Notably, this improvement in survival was achieved without any discernible impact on the severity of toxicity [169]. A subsequent meta-analysis comparing RT alone with RT + HT corroborated these findings. A synthesis of the eight included studies revealed an odds ratio (OR) of 2.67 (95% CI: 1.57–4.54, *p* < 0.001) for CR and an OR of 2.61 (95% CI: 1.55–4.39, *p* < 0.001) for locoregional control. Moreover, the network meta-analysis incorporated within the present study further substantiated the efficacy of augmenting HT with triple therapy using CHT [171]. A meta-analysis evaluating various treatment techniques for locally advanced cervical cancer also demonstrated that adding HT optimizes treatment outcomes [170]. Despite the endorsement of this combined treatment, a substantial proportion of clinical trials have failed to demonstrate a substantial superiority of HT as an addiction treatment. However, there is still a paucity of head-to-head comparisons among studies involving RT, RT+HT, and RT+HT+CHT.

### Pelvic tumors- rectum, bladder, prostate cancers

The majority of studies examining the use of RT with HT in prostate cancer have focused on advanced or recurrent cancers [172–174, 176, 177]. The predominant technique employed in these settings was EBRT, with HT administered either locally with transrectal ultrasound or regionally via radiofrequency. A particular study assessed the efficacy of this approach in patients experiencing biochemical recurrence of prostate cancer [178]. In a seminal phase I/II clinical trial, 12 patients with locally advanced prostate cancer were enrolled. The treatment regimen comprised definitive RT in doses ranging from 65 to 70 Gy, along with HT sessions administered once or twice a week, with a target temperature of at least 42.5 °C for the prostate gland. The targeted temperature was achieved in only 3.5% of the HT sessions. The 3-year disease-free survival (DFS) rate was 68% in the HT group, while the control group exhibited a rate of less than 50% [172]. In a separate study by Fosmire et al., a novel method of delivering heat via transrectal ultrasound was employed. This approach resulted in an acceptable toxicity profile, with only two patients experiencing treatment-limiting toxicities [173]. Another method of HT use was interstitial HT with cobalt-palladium thermoseeds, performed once a week, combined with an EBRT dose of 68.4 Gy in 38 fractions, which also had a favorable toxicity profile and reduced prostate-specific antigen (PSA) levels from 11.6 to 0.55 ng/ml two years after the therapy [175]. Concurrent studies have demonstrated comparable outcomes with combined treatment regimens, accompanied by substantial declines in PSA levels post-therapy [177, 178]. However, a discerning evaluation of these findings is warranted, given the heterogeneity of the techniques employed and the preponderance of studies conducted during the early 2000s (Table 1). Currently, further clinical trials are being conducted to validate these results, including HETERERO NCT04889742 and NCT03238066.

The majority of studies on bladder cancer employ a combination of three or more therapeutic modalities. A study was conducted that compared thermochemoradiotherapy with chemoradiotherapy. The study included 46 patients with invasive bladder cancer. RT was administered at a dose of 1.8–2.0 Gy daily or 2.0–4.0 Gy twice weekly, preceded by 60 minutes of HT at 42.5 degrees Celsius. In addition to this, patients received CHT based on adriamycin or cisplatin. The ORR was found to be 43.5% [194]. The phase IIB study, in addition to the aforementioned regimens, incorporated transurethral resection of bladder tumors. Patients with T2-4 muscle-invasive bladder cancer received sequential surgery, R at doses of 57–58.2 Gy with concurrent weekly platinum-based CHT and weekly deep HT (41–43 °C, 60 min). The study achieved a 93% CR rate and 2-year local recurrence-free survival (LRFS) and OS of 81 and 86%, respectively [195]. The aforementioned study by van der Zee et al., which included patients with bladder cancer, also demonstrated a significant benefit from the addition of HT, particularly in terms of ORR and OS [164]. These findings suggest that HT may also be beneficial in this context; however, it is important to note that these studies are of a distant nature and that further evidence from randomized prospective trials is necessary to validate these results.

RT has been employed in the treatment of rectal cancers for years, but the treatment paradigm has undergone significant changes in the last year. According to the latest guidelines of the American Society for Radiation Oncology (ASTRO), intensification of preoperative treatment is recommended in order to perform less invasive procedures and preservation of organs [196]. HT has emerged as a promising modality for achieving downstaging in these patients. A study of 28 patients (T2 or T3 rectal adenocarcinoma in the lower rectum, T4 rectal cancer in the middle or upper part of the rectum) with preoperative RT of 40 or 50 Gy and concurrent weekly whole pelvis HT and CHT based on 5-FU and leucovorin demonstrated beneficial effects. The study observed that downstaging was achieved in 41.4% of all cases and 52.6% of cases that received 50 Gy RT [197]. Another phase 2 study, which incorporated metronidazole into HT, demonstrated that following treatment, R0 resection was attained in 92.2% of patients, and the 2-year OS and DFS were 91 and 83%, respectively [198]. The most recent phase II study (NCT02353858) yielded analogous results, with the majority of patients demonstrating a pathological response to treatment in the post-surgical setting. The 3-year OS and DFS rates were 94 and 81%, respectively [199]. A meta-analysis was conducted to summarize the effects of combining chemoradiotherapy with deep regional HT in patients with rectal cancer, and the results were encouraging. The study incorporated data from patients diagnosed with locally advanced rectal cancer and locally recurrent rectal cancer, encompassing a total of 782 cases. The pooled 5-year OS rate among patients was 87% (95% CI: 83–90%). However, given the considerable heterogeneity in the utilization of HT in the included studies and the moderate quality of the reporting, it is challenging to derive consistent conclusions for the entire group [200].

### Sarcomas and melanoma

The clinical trial data on the combination of RT and HT in these diagnoses is limited. To date, there is only one clinical trial with melanoma [182] and four studies on soft tissue sarcomas (STS) (Table 1) [9, 179–181]. In the context of STS, the combination of HT with CHT has demonstrated efficacy [201, 202]. However, the available clinical trials indicate that RT+HT therapy is effective and is associated with relatively minor side effects; nevertheless, further investigation is necessary to ascertain its full safety and efficacy profile. Presently, studies are underway to assess the efficacy of combining various RT techniques with HT in STS. Notably, a phase I/II clinical trial (HYPROSARC; NCT01904565) is currently ongoing, which involves the concurrent administration of local HT and proton beam radiotherapy in primary or recurrent unresectable STS of the extremities, trunk, or retroperitoneum. Additionally, a phase II trial (HOT; NCT04398095) is underway to investigate the effectiveness of hypofractionated radiotherapy with deep HT in recurrent and radiation-induced STS and bone sarcomas.

### Head and neck region tumors

The findings from the collaborative RT and HT investigations in head and neck cancers (HNC) are promising. Several randomized clinical trials have been published in recent years on this subject (Table 1) [183–188]. HT appears to be effective in this context, particularly in cases of locally advanced disease with regional lymph node involvement [187, 188]. Reports have emerged of re-irradiation in combination with HT and CHT, and despite the intensification of treatment in these cases, no significant deterioration in the safety profile or acute toxicities was observed. However, due to restrictions imposed by the ongoing pandemic, poor compliance resulted in a paucity of reports of efficacy. Notably, this study employs a distinct HT technique, fever-range whole-body HT [183]. Two of the clinical trials also assessed specifically nasopharyngeal cancers, in both of which the combination was clearly superior to RT as a single treatment method. One trial demonstrated CR rates of 81.6% with HT, which was significantly superior to the 62.8% observed in the control group (*p* = 0.014) [184]. Another study reported CR rates of 95.6% in the HT group and 81.1% in the control arm (*p* = 0.003) [185]. A subsequent meta-analysis encompassing six studies involving 451 patients diagnosed with HNC demonstrated that HT+RT augments the probability of CR by approximately 25% in comparison with RT alone. The study made a comparison of the CR rate subsequent to the therapeutic intervention. In the cohort of patients undergoing treatment with HT+RT, the CR rate was documented to be 62.5% (range: 33.9–83.3%), while in the group subjected exclusively to RT, the rate was recorded at 39.6% (range: 31.3–46.9%) (risk ratio: 1.61, 95% CI: 1.32–1.97, *p* < 0.0001). Furthermore, acute and late grade III/IV toxicities were reported to be similar in both groups [7]. Considering these results, further research on HNCs appears reasonable.

### Other indications

Moreover, there are also reports from studies on diagnoses in which HT is used with conventional therapies less frequently. HETPAC (NCT02439593) is a randomized phase 2 clinical trial that evaluates thermochemoradiotherapy in comparison to chemoradiotherapy in patients with inoperable, locally advanced pancreatic cancer. The study design anticipates that patients will receive four courses of neoadjuvant CHT according to the FOLFIRINOX regimen, followed by randomization. The control group will then receive RT with gemcitabine and the study group RT with HT. The RT regimen involves the delivery of 56 Gy and 50.4 Gy to the gross and clinical target volumes, respectively, in 28 fractions. In the HT arm, the treatment will be administered weekly at temperatures ranging from 40 to 43 °C for a duration of 60 minutes. The primary endpoint of this study is the improvement of OS at one year from 40% to 60% compared to the control arm. The results of this study are currently pending [203]. A separate study on pancreatic cancer demonstrated a mOS of 15 months in the HT group compared to 11 months in the control group, yielding a statistically significant result (*p* = 0.025) [204].

Another phase II trial examined the effectiveness of HT combined with RT and trans-arterial chemo-embolization (TACE) among patients with hepatocellular carcinoma and portal vein tumor thrombosis. Following TACE, 10 fractions of 3-5 Gy were administered concurrently with HT. The 3-month evaluation revealed an ORR of 69.6%. However, 80% of patients experienced pain after HT, and 13 patients refused further treatment [205]. The findings concerning non-small cell lung cancer (NSCLC) remain equivocal. A study involving 80 patients with locally advanced NSCLC treated with RT and HT revealed no significant differences in terms of OS or local response to treatment. However, the HT group exhibited a superior LPFS (1-year LPFS 67.5% vs. 29%, *p* = 0.036) [108]. Another study demonstrated that the outcomes observed in patients are contingent on the parameters of the treatment modality employed [193]. A study on recurrent gastric cancer, in addition to the outcomes of the effectiveness of the addition of HT, also indicated changes in the immune system of patients. The study population was segmented into two groups, with 86 patients assigned to each group. One group underwent RT as a standalone treatment, while the other group received a combination of abdominal HT. The study’s outcomes revealed a significantly higher response rate in the HT group compared to the control group (57.2% vs. 47.1%, *p* = 0.04). Further analysis revealed that patients in the HT group exhibited enhanced levels of CD3+, CD4+, and NK cells, along with an increased CD4+/CD8+ ratio. In contrast, no significant changes were observed in the control group [206]. A study of esophageal cancer has been conducted in which the use of preoperative radiochemotherapy combined with HT vs. RT alone was compared. The 3-year OS rates in the HT group and the control group were 67.4% and 41.8%, respectively. However, it is difficult to conclude whether the benefit was due to HT or the combined treatment with CHT [207]. Conversely, a meta-analysis that evaluated the efficacy of HT in advanced oesophageal carcinoma combined with chemoradiotherapy demonstrated improvements in long-term and short-term curative effects. A total of 1,519 patients were evaluated, and the 1-, 3-, 5-, and 7-year OS rates exhibited a significant preference for the incorporation of HT (*p* < 0.05). However, no enhancement was observed in terms of recurrence rate and distant metastasis rate [208].

Contemporary methods of HT administration, employing nanoparticle delivery intratumoral, are now emerging. For instance, in glioblastoma, the use of these heatable particles with RT of a total dose of 30 Gy, fractionated at 2 Gy, resulted in a mOS following diagnosis of first tumor recurrence of 13.4 months (95% CI: 10.6–16.2 months) [209].

A novel and promising application of HT in the palliative treatment of metastatic foci has emerged from recent studies, which have demonstrated the efficacy of combined treatment modalities in achieving enhanced pain management and local response of bone metastases. In the initial study, the administration of RT at 30 Gy in 10 fractions in conjunction with HT resulted in a median time to pain progression of 55 days in the RT-only group and was not attained in the HT group (*p* < 0.01). The response rate was higher in the RT-HT group [210]. The second phase III study employed the same fraction and total doses of RT but utilized whole-body HT. The results of this study were similar to those of the first study, as the CR rate for HT was 47.4% versus 5.3% within two months post-treatment (*p* < 0.05). Additionally, the time to pain relief was 10 days in the HT arm and not reached in the control arm [211]. This finding suggests the potential for a beneficial effect of whole-body HT as well as local HT in addressing this specific indication.

## Safety and adverse events

The incorporation of supplementary modalities into oncology treatment regimens frequently results in elevated toxicity levels within these regimens. Consequently, a primary concern in contemporary radiation treatment is the potential for heightened AEs that may be induced by the addition of HT. As demonstrated in the preceding chapter, studies have shown that HT is both effective and safe when used as an adjunct to therapy. While clinical studies have identified a wide range of possible adverse effects, they have not yet been directly attributed to HT. Instead, these effects often result from the use of RT and are typical for this type of treatment. A direct examination of the adverse effects of HT in the treatment of cervical cancer revealed that no patient had to discontinue treatment due to such effects, and no grade IV AEs were observed, with grade III occurring in 8% of patients. The predominant complaint reported during therapy was a feeling of weakness, and a notable observation was the occurrence of AEs earlier in the group of patients treated with RT+HT than in those treated with RT alone [168]. A further study on cervical cancer also found no significant difference in early and late toxicity between the HT and control groups, primarily grade 0–1 AEs, with 6% of patients experiencing grade II bladder AEs [156]. These results are consistent with most studies on cervical cancer. A subsequent investigation focused on breast cancer likewise determined there were no substantial AEs attributable to the incorporation of HT, and no statistically significant variation in ≥grade 2 toxicity among heated versus control groups was observed (*p* = 0.38). Furthermore, no association was observed between the number of HT sessions and toxicity (*p* = 0.09) [146]. Despite employing a more invasive approach, with the utilization of transrectal ultrasound HT, no intensification of AEs was observed. The level of tolerance exhibited a favorable response in 17 out of 22 cases, a satisfactory response in 3 out of 22, and a treatment-limiting response in 2 out of 22 cases. The treatments that resulted in position intolerance and/or pain, as well as hematuria, occurred in 5 out of 22 patients who received treatment [173]. A phase II study in prostate cancer, the primary endpoints of which were the rate of acute genitourinary, gastrointestinal, and HT-related toxicities, revealed that no grade ≥3 toxicities occurred. HT-specific symptoms of grades 2 and 3 were observed in 4 and 2% of the patients, respectively [178]. Whole-body HT did not result in an increase in acute toxicity. However, the study was conducted with a limited sample size of five patients, and it was prematurely terminated due to inadequate compliance [183]. In light of these findings, it appears that HT is generally well-tolerated by patients, with no reported serious AEs. It is noteworthy that the majority of reported side effects associated with HT are limited to moderate to severe pain and discomfort, as well as an unpleasant sensation of high heat. These symptoms manifest on average in 20–40% of patients, yet do not adversely affect the therapeutic course or outcomes [9, 145]. The safety of HT in combination with standard CHT-based treatment appears to have been demonstrated. In consideration of future advancements in treatment modalities, it is imperative to assess the efficacy of RT+HT in conjunction with contemporary systemic treatment regimens, encompassing immunotherapy and molecularly targeted therapies.

## Summary

In recent years, significant progress has been made in understanding the possibilities of combining RT with HT in oncological treatment. The underlying mechanisms of the synergy between these two techniques in inducing cellular damage in cancer cells and stimulating the immune system of patients have been recognized for years. Preclinical studies have indicated potential avenues for the utilization of HT in clinical settings. However, there are still issues that require broader understanding. In order to maintain an optimal balance between synergistic effects such as the induction of DNA damage by RT and the inhibition of its repair by HT, it seems necessary to develop the most effective temperature range, the duration of heat-induced vasodilation affecting thermotolerance, and the pathophysiology underlying CSC radiosensitization by HT. Additionally, the occurrence of an abscopal effect and the precise mechanism of immune stimulation remain to be fully elucidated, as this effect is only discernible in a very small percentage of patients [104, 117, 118, 212]. The majority of clinical trials have demonstrated the efficacy of incorporating HT into standard treatment regimens, yielding superior outcomes without an escalated incidence of therapy-related adverse events. However, it is important to note that the majority of the clinical trials that were discussed were conducted during the 1990s. This introduces a significant bias, as advances in both RT and HT techniques, as well as the paradigm shift from classical systemic chemotherapy to immunotherapy and molecularly targeted therapy, are currently introducing completely new conditions for the use of HT. While the theoretical framework suggests that enhancements in systemic treatment, when integrated with HT, should yield optimal outcomes, not all indications have sought to validate the safety of this combination. Furthermore, the existing studies have been marked by significant heterogeneity, with HT frequency ranging from once weekly to daily applications and mean achieved temperature profiles diverging widely between 39 and 43 °C. In trials evaluating HT specification, higher temperatures or HT frequencies were associated with better outcomes, implying the need for unification. Also, the results reported from the studies are heterogeneous. Some of the studies discuss the local benefit of the addition of HT, some focus on ORR and responses to radical treatment, and some discuss the results in a long-term context, focusing on OS values. Due to the varying locations, the description of AEs from the aforementioned studies also differs. These factors have the potential to influence the overall conclusion.

Recent advancements in the field include novel techniques in RT and HT, as well as their potential combination. There are reports of studies combining HT with ITH, demonstrating the safety and effectiveness of such a combination. In the phase I study with HT, adoptive cell therapy and CHT or anti-PD-1 antibodies, the majority of patients experienced mild adverse effects. Additionally, beneficial changes in the immune profile of the patients were observed, expressed by an increase in the number of proinflammatory cytokines [95]. Research on optimal thermoradiotherapy planning is underway [122], and studies have demonstrated that simultaneous planning allows for the delivery of a dose equivalent to 10 Gy by HT, which has been shown to inhibit DNA repair in irradiated cells [5]. Other reports have indicated the optimization of reoxygenation 24–48 hours after HT application, suggesting a more favorable clinical effect [213]. However, it should be noted that not all patients respond in the same way, and further studies on physiological-temperature dependencies are necessary. An interesting concept recently developed in research is the delivery of HT using precise nanoparticles [214]. This method enables precise delivery of therapy to a predetermined area, with the induction of heat at the scale of these particles resulting in significant biological effects, including necrosis, DNA damage, and immune stimulation, directly within the tumor [215]. Furthermore, nanoparticle delivery has the potential to be effective in tumor areas with significant hypoxia, where conventional HT methods have been ineffective [216]. Recent advancements in RT have also been substantial, with reports emerging on the combination of proton-based radiation treatment with HT [217]. A notable study on uveal melanoma demonstrated the efficacy of this combination, as the degree of enucleation after treatment was significantly reduced [218]. In summary, although the preliminary demonstration of the significant utility of incorporating HT into radiation treatment protocols is clear, the significant developments in both of these techniques and the significant unknowns regarding the induction of biological effects force us to continuously develop new randomized clinical trials using newer methods with the introduction of currently available techniques into routine clinical practice in order to find the exact place for such a procedure.

## Data Availability

No datasets were generated or analysed during the current study.
